# Harvestmen of the BOS Arthropod Collection of the University of Oviedo (Spain) (Arachnida, Opiliones)

**DOI:** 10.3897/zookeys.341.6130

**Published:** 2013-10-07

**Authors:** Izaskun Merino-Sáinz, Araceli Anadón, Antonio Torralba-Burrial

**Affiliations:** 1 Universidad de Oviedo - Dpto. Biología de Organismos y Sistemas, C/ Catedrático Rodrigo Uría s/n, 33071, Oviedo, Spain; 2 Universidad de Oviedo - Cluster de Energía, Medioambiente y Cambio Climático, Plaza de Riego 4, 33071, Oviedo, Spain

**Keywords:** Opiliones, Arthropoda, Iberian Peninsula, entomological collections, biodiversity collections, distribution, datasets, Spain, Portugal

## Abstract

There are significant gaps in accessible knowledge about the distribution and phenology of Iberian harvestmen (Arachnida: Opiliones). Harvestmen accessible datasets in Iberian Peninsula are unknown, an only two other datasets available in GBIF are composed exclusively of harvestmen records. Moreover, only a few harvestmen data from Iberian Peninsula are available in GBIF network (or in any network that allows public retrieval or use these data). This paper describes the data associated with the Opiliones kept in the BOS Arthropod Collection of the University of Oviedo, Spain (hosted in the Department of Biología de Organismos y Sistemas), filling some of those gaps. The specimens were mainly collected from the northern third of the Iberian Peninsula. The earliest specimen deposited in the collection, dating back to the early 20^th^ century, belongs to the P. Franganillo Collection. The dataset documents the collection of 16,455 specimens, preserved in 3,772 vials. Approximately 38% of the specimens belong to the family Sclerosomatidae, and 26% to Phalangidae; six other families with fewer specimens are also included. Data quality control was incorporated at several steps of digitisation process to facilitate reuse and improve accuracy. The complete dataset is also provided in Darwin Core Archive format, allowing public retrieval, use and combination with other biological, biodiversity of geographical variables datasets.

## General description

**Figure 1.**
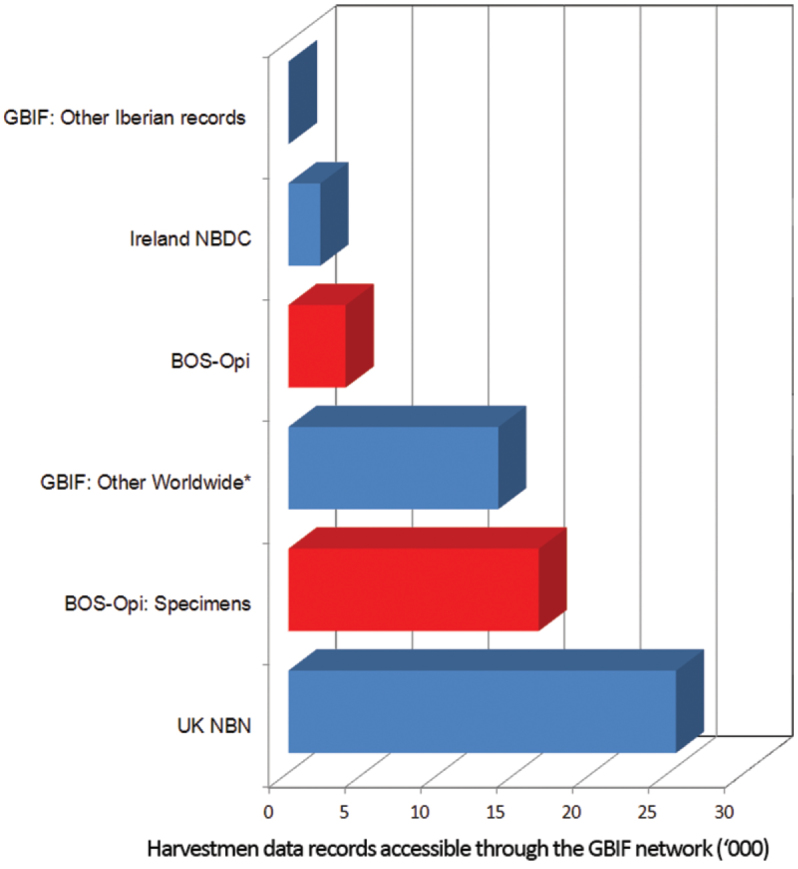
BOS-Opi contributes significantly to the publicly accessible Harvestmen data records through the GBIF network.

**Purpose:** Existing knowledge on the distribution of harvestmen in the Iberian Peninsula is still very fragmented (Prieto 2003). There are biodiversity collections with more data on Iberian harvestmen, both in terms of numbers of specimens and of localities; these records are partly published for some genera (e.g., Prieto 2004, Prieto and Fernández 2007, Merino-Sáinz et al. 2013a). However, there is no dataset that allows public retrieval or use these data. Thus, only 48 records of Iberian harvestmen are available in GBIF [http://data.gbif.org, accessed on 03 July 2013: Museum of Comparative Zoology Harvard University 43 records; Museum of Zoology University of Navarra MZNA 3 records; Senckenberg Collection Arachnology SMF 2 records]. Only two other datasets in GBIF are composed exclusively of harvestmen records: the Opiliones dataset of the UK National Biodiversity Network (http://data.gbif.org/datasets/resource/854, based on Sankey 1988 and Hillyard 2005), which includes 25,486 records, and the Harvestmen (Opiliones) of Ireland dataset of the National Biodiversity Data Centre (http://data.gbif.org/datasets/resource/10810), with 2,109 records (there are apparently additional 13,800 harvestmen records in GBIF from several datasets comprising more taxonomic groups) (Figure 1).

The purpose of this paper is to document a dataset corresponding to Opiliones specimens deposited in the BOS Arthropod Collection (subcollection of Opiliones: BOS-Opi) of the University of Oviedo, Spain, comprising 16,455 specimens in 3,772 vials (each vial containing specimens with the same species/locality/date/capture method information, i.e., a single record). As a result of this, the BOS-Opi dataset makes a significant contribution of primary data about Iberian harvestmen for ecological, faunistic and conservation studies. With the publication of this dataset, we aim to (1) providing a dataset with phenological and distribution data on harvestmen from the northern third of the Iberian Peninsula, and (2) describing the Opiliones subcollection of the BOS Arthropod Collection.

**Additional information:** A list of publications citing harvestmen contained in this dataset (BOS-Opi) is provided in point 2 of the reference section.

## Project details

**Project title:** Informatización de la Colección de Artrópodos BOS de la Universidad de Oviedo / Digitisation of the BOS Arthropod Collection of University of Oviedo

**Personnel digitisation:** Torralba-Burrial A

**Administrative contact:** Anadón A

**BOS-Opi determination specialist:** Merino Sáinz I

**BOS-Opi collectors:** Collectors who have deposited more than 50 specimens include Merino Sáinz I, Anadón A, Fernández-Álvarez F.A., Torralba-Burrial A, Ocharan Larrondo FJ, Melero Cimas VX, Monteserín Real S, Ocharan Ibarra R, Rosa García R, and Vázquez Felechosa MT

**Curator of *P. Franganillo Collection*:** Lastra C

**Funding:** Digitisation of this biological collection was supported by the Spanish National R+D+i Plan (MICINN, Spanish Government, grant ref. PTA2010-4108-I) and PCTI Asturias (Asturias Regional Government, ref. COF11-38) through a contract for ATB.

Almost 73% of the specimens were collected as part of the PhD Thesis by Merino Sáinz (2012), which was supported by a Severo Ochoa pre-doctoral grant (ref. BP08039, FICYC, Asturias Regional Government). The project entitled “Cataloging Biodiversity of Muniellos Biosphere Reserve” was supported by the Asturias Regional Government (ref. SV-PA-00-01, SV-PA-01-06, SV-PA-02-08 and SV-PA-03-13).

**Figure 2.**
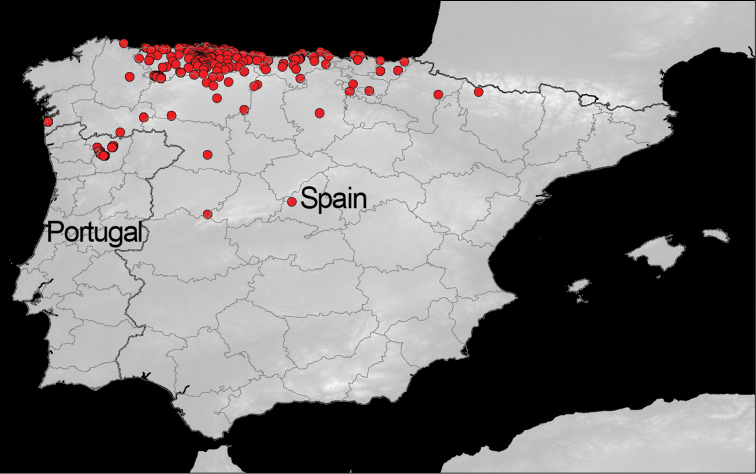
BOS-Opi facilitates access to harvestmen specimen data collected from northern region of Iberian Peninsula.

**Study area description:** Harvestmen specimens deposited in BOS Arthropod Collection are from the northern third of the Iberian Peninsula (Figure 2). Most of this zone belongs to the Atlantic bioregion (from the Cantabrian Mountains to the Cantabrian Sea), with the Mediterranean bioregion in the south (the biogeographic regions are based on vegetation types as described by Rivas-Martínez et al. (2004) and European Union Habitats Directive 92/43/CEE). The Atlantic/Eurosiberian bioregion (from which the majority of specimens were collected) is a more humid zone with less summer drought compared to the Mediterranean bioregion (Rivas-Martínez 1987, AEMET and IM 2011). The climatic and habitat conditions also vary within this bioregion depending on the orography and geology (calcareous/siliceous) of the area (Rivas-Martínez 1987, AEMET and IM 2011). Oak and beech forests are the main potential vegetation in the area, but significant anthropogenic modifications have reconfigured the landscape throughout much of the territory (e.g., Díaz González and Prieto 1994). Harvestmen communities, as components of soil biodiversity, have an important role to play in the assessment of the mosaic of agricultural landscapes from the northern part of the Iberian Peninsula (e.g., Rosa García et al. 2010, 2011, Merino Sáinz 2012, Merino-Sáinz et al. 2013b).

**Design description:** The digitisation process of this dataset (BOS-Opi) was carried out according to the workflow put in place for the Odonata subcollection (BOS-Odo) (Torralba-Burrial and Ocharan 2013). Prior to digitisation, the preservation status of each specimen is evaluated and enhanced, and then a taxonomic identification with suitable literature is made (or reviewed when pre-existing) by a specialist. For reasons of optimizing storage of specimens in the collection, harvestmen specimens collected from same species, locality, date and capture method (i.e., a “record”) are kept in the same vial. Digitisation of biodiversity data and retrospective georeferencing are then carried out. Best practices as suggested by Chapman (2005a) and Chapman and Wieczorek (2006) are followed for the georeferencing processes. Digital cartography (the gazetteer IBERPIX v2) was used for georeferencing. All data associated to specimens is managed with ZOORBAR software. The dataset is exported to DarwinCore v1.2 format and uploaded to the IPT of the GBIF Spanish node (http://www.gbif.es:8080/ipt). DarwinCore elements included in the dataset structure are listed in the dataset description section. Data quality controls of geographic, taxonomic and additional data associated with the harvestmen specimens were performed at several steps of digitisation process as an essential part of this Information Management Chain (Chapman 2005a, 2005b), as detailed in Torralba-Burrial and Ocharan (2013); these are explained in the quality controls section below.

Currently, dataset is being used to study phenological and life history differences of harvestmen species between areas in north Iberian Peninsula with different geographical/habitat features, species distribution and importance of opportunistic data in fill knowledge gaps when standardised sampling data are not available or are incomplete. Moreover, this dataset is considered as a dynamic catalogue of the harvestmen of BOS Arthropod Collection, allowing free access of citizens, researches, environmental companies and government managements to biodiversity data kept in this Collection.

## Taxonomic coverage

**Figure 3.**
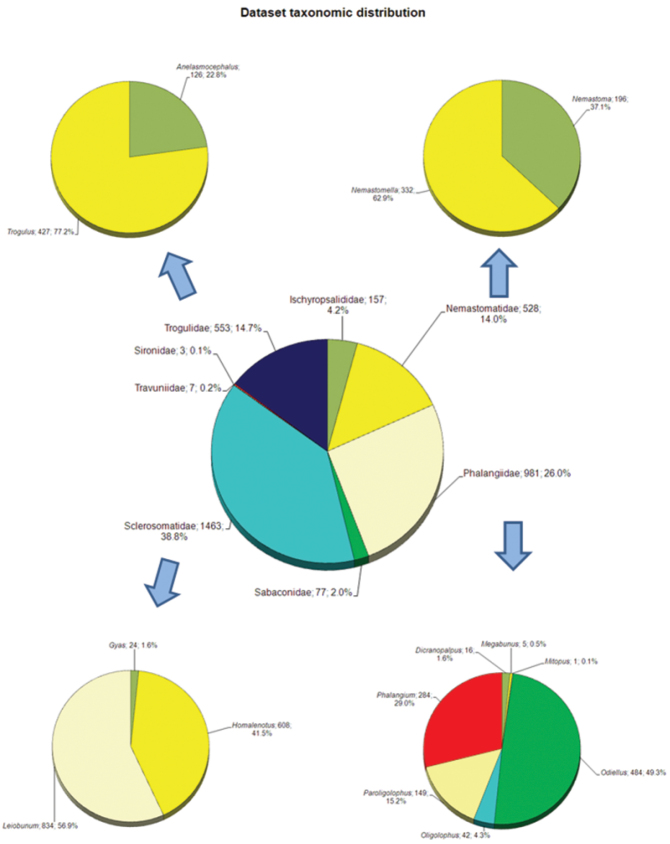
Taxonomic coverage of the BOS-Opi dataset.

**General taxonomic coverage description:** All specimens were identified to species when preservation status, sex and life cycle phase permitted it. Sixty-two species were recorded from the northern third of the Iberian Peninsula (Merino Sáinz and Anadón 2008), 23 of which are included in this dataset (most of the absent species are from cave and subterranean habitats, difficult to found without specific samples). There are ten harvestmen families recorded from the Iberian Peninsula, and eight of these are represented in this dataset. Only Dicranolasmatidae (suborder Palpatores) and Phalangodidae (suborder Laniatores) are missing. As depicted in Figure 3, the family with the largest number of specimens in the collection is Sclerosomatidae (38.82%, consisting of the genera *Leiobunum*, *Homalenotus* and *Gyas*), followed by Phalangiidae (26.0%: *Odiellus*, *Phalangium*, *Paroligolophus*, *Oligolophus*, *Dicranopalpus*, *Megabunus* and *Mitopus*), Trogulidae (14.7%: *Trogulus* and *Anelasmocephalus*), Nemastomatidae (14.0%: *Nemastomella* and *Nemastoma*). Other families represent less of 5% of the records (Figure 3).

No types are hosted among the Opiliones of the BOS Arthropod Collection. However, this collection does include the historic *Collection of Arachnids P. Franganillo*, with 17 specimens (in ten vials with BOS-Opi codes 3758-3767, five missing since the cataloguing of the collection by Lastra 1974) from the early 20^th^ Century. Pelegrin Franganillo published many new species of arachnids during the first quarter of the century, with very short (if any) descriptions and without figures. In four publications Franganillo (1913, 1917, 1925, 1926) cited, described or commented on Iberian harvestmen. The location of the collection was unknown since the death of Franganillo (in 1955 at La Habana, Cuba) and no comparison with type specimens was possible. For these reasons, most of the names given by Franganillo are considered as *nomina dubia* both in Araneae (e.g., Urones 1996, Duncan et al. 2010, Crews 2011) and in Opiliones (e.g., Prieto 2003), are synonymized (e.g., Kraus and Kraus 1988, Alayón García 2002, Polotow and Brescovit 2009, Miller et al. 2012), or his records were discarded when other cross-checking sources were not available (e.g., Cardoso and Morano 2010). In 1972, part of the Spanish collection of P. Franganillo was found in a garret of the “La Inmaculada School” (where Franganillo was a teacher) and J.M. Patac de las Traviesas donated it to Oviedo University (see Lastra 1975). The preservation status was very deficient: specimens were dried and locality/determination labels were missing, but almost all vials had a collection number, and assignation and reconstruction of the collection catalogue (without localities or type assignation) was possible for the vials present (Lastra 1974). A study of this collection reveals misidentifications of other species in Araneae (Méndez 1998). In Opiliones, species described by Franganillo are considered *nomina dubia* (Prieto 2003), and most of his records of other species have been discarded (Mello-Leitao 1936, Prieto 2003). Identifications of the harvestmen specimens of the Franganillo collection at the University of Oviedo show previous misidentifications (*Oligolophus vittiger* Simon is an *Odiellus* sp., two specimens of *Phalangium parietinum* de Geer are really *Gyas titanus* Simon), current identifications more accurate (three specimens of *Liobunum* sp. belong to *Leiobunum blackwalli* Meade) and other specimens show a correct identification by Franganillo (*Phalangium opilio* Linnaeus).

## Taxonomic ranks

Kingdom: Animalia

Phylum: Arthropoda

Class: Arachnida

Order: Opiliones

Family: Ischyropsalididae, Nemastomatidae, Phalangiidae, Sabaconidae, Sclerosomatidae, Sironidae, Travuniidae, Trogulidae.

**Common names:** Animals, Arthropods, Arachnids, Harvestmen.

## Spatial coverage

### General spatial coverage

All specimens are from the northern part of the Iberian Peninsula (Figure 2). Most of them are from Asturias province (89.58% of records with “species/locality/date”), with other specimens originating from Cantabria (6.86%), Tras-os-Montes (1.20%), Pontevedra (0.88%) and other provinces (León, Burgos, Álava, Guipúzcoa, Vizcaya, Lugo, Palencia, Ourense, Zamora, Huesca, Salamanca, Navarra and Madrid).

### Coordinates

40°18'N and 43°42'N Latitude; 8°54'W and 0°30'W Longitude.

### Temporal coverage (specimens’ data range)

1900–2012

### Temporal coverage (collection formation)

1977-present

## Natural collections description

**Parent collection identifier:** Colección de Artrópodos BOS

**Collection name:** Colección de Artrópodos BOS de la Universidad de Oviedo: Opiliones (BOS-Opi)

**Collection identifier:** http://data.gbif.org/datasets/resource/15038

**Specimen preservation method:** Ethanol 70°

**Curatorial unit:** 3772 with an uncertainty of 0 (Vials (records))

**Curatorial unit:** 16455 with an uncertainty of 0 (Specimens)

## Methods

**Method description:** The digitisation process of the Opiliones subcollection (BOS-Opi) was realised in accordance with the published workflow of the Odonata subcollection (BOS-Odo) (see Torralba-Burrial and Ocharan 2013).

*Pre-digitisation phase*: The preservation status of harvestmen specimens was reviewed prior to digitisation. Vials were changed when necessary and refilled with preservation liquid (ethanol 70°). Specimens were identified or identifications were reviewed when they were already noted. Identification labels were added when labels were lacking or otherwise incomplete. Specimens’ vials were sorted alphabetically by family/genus/species names in trays, and hosted in metallic cabinets in a cold chamber.

*Digitisation phase*: A database with DarwinCore v1.2 standard fields and other fields specific to different research projects was developed using MS EXCEL software. All biodiversity data available on the specimens’ labels (i.e., specimen code, species identification and name of determiner, sex, number of specimens in the vial, locality, date, habitat, collector, collection method, research project and observations) were included in the database.

Other geographic data (municipality, GPS coordinates, altitude, etc.) from specimen labels or from associated publications were added to the database when available. If coordinates were not present on the specimen labels or in primary publications, retrospective georeferencing (see Chapman and Wieczorek 2006) was carried out using digital cartography tools (mainly the public gazetteer IBERPIX v2, compiled by the Spanish National Geographic Institute, http://www.ign.es/iberpix2/visor). Localities were sorted geographically for batch retrospective georeferencing, starting with larger batches (Chapman and Wieczorek 2006). Coordinates were stored in MGRS format, and IBERPIX v2 was used to calculate the uncertainty radius of the place georeferenced.

The database was converted and imported to, and managed with, ZOORBAR v2.1.1 software (Pando et al. 1996–2012).

*Creation of the dataset*: The dataset was exported as a file in DarwinCore v1.2 format and geographic coordinates were carried out with ZOORBAR v2.1.1 software. DarwinCore elements included in dataset structure are listed in the dataset description section. Data format, georeferenced coordinates and absence of ASCII anomalous characters were checked with DARWIN_TEST v.3.2 software (http://www.gbif.es/darwin_test/Darwin_test.php). Erroneous data were corrected and data cleaning was repeated to enhance the data quality (see details in the section on quality control).

The dataset was transformed to a DarwinCore Archive format with metadata to ensure rapid discovery of this biodiversity resource and future publishing as a citable academic paper (see Chavan and Penev 2011). The dataset was uploaded to the Integrated Publishing Toolkit (IPT v2.0.4) Platform of the Spanish node of the Global Biodiversity Information Facility (GBIF) (http://www.gbif.es:8080/ipt). Links to these data were also provided on the BOS Arthropod Collection website (http://www.unioviedo.es/BOS/Zoologia/artropodos). The offline version of the dataset includes the identification history of each specimen (4149 items), collection method, research project, and notes on materials derived from the specimens (e.g., publications). This information is available on request.

**Figure 4.**
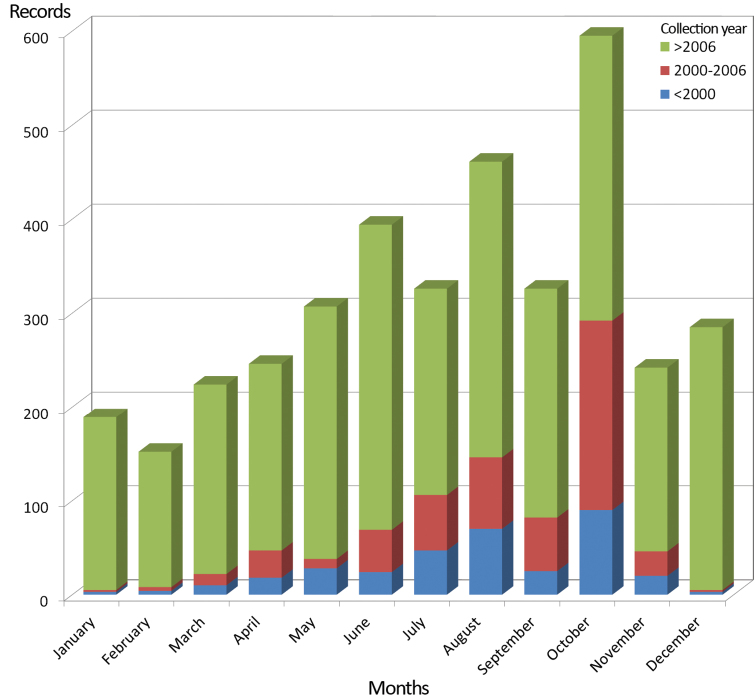
Cumulative monthly distribution of the records of BOS-Opi dataset.

**Study extent description:** Specimens are mainly from the northern third of the Iberian Peninsula (see geographic coverage section). The earliest specimens are from the 20^th^ century (belonging to the P. Franganillo collection), but the general collection starts in 1977. However, only 9.73% of the items were collected prior to the year 2000, while 75.93% were collected between 2009 and 2012. The BOS-Opi dataset includes the record distributions by month (cumulative number of records in Figure 4), in several cases stemming from repeated sampling in each locality; this information is useful for studies of the life cycles of harvestmen from the region (e.g. Merino Sáinz 2012) and for making comparisons with other regions.

**Sampling description:** Material deposited in the Opiliones subcollection of the BOS Arthropod Collection has been collected in three ways:

1) specimens from the PhD dissertation by Merino Sáinz (2012) carried out at the University of Oviedo (72.99% of items);
2) specimens from the project “Cataloguing of the Biodiversity from the Biosphere Reserve of Muniellos” (SW of Asturias province) (Ocharan Larrondo et al. 2003) (13.10%);
3) specimens from other sources: collections from students in Biology and Forestry Engineering programs at the University of Oviedo, other research projects, practical courses, etc. (13.92%).

Most of the specimens were collected with pitfall traps (85.15%). Ethylene glycol was used as a fixation and preservation liquid in the pitfalls (proven effective in various environments and for taxonomic groups including arachnids; Schmidt et al. 2006, Jud and Schmidt-Entling 2008, Cheli and Corley 2010). Sodium polyphosphate was added to reduce surface tension and to facilitate the capture of arthropods. Direct (hand) collection of specimens and sweep netting yielded 14.26% and 4.32% of the specimens, respectively. Other methods combined (vegetation beating over an upturned umbrella, Berlese funnel, light trap, Malaise trap, sieve) yielded a much lower number of specimens (1.2%) (see Barrientos 2004, Merino Sáinz 2012 for descriptions).

**Quality control description:** Validation and cleaning of geographic, taxonomic and additional data associated with the harvestmen specimens was incorporated at several steps of the process as an essential component of the digitisation project (see Chapman 2005a, b). Workflow was similar to the one described by Torralba-Burrial and Ocharan (2013). Specimens were identified or else their identification revised by an expert (I. Merino-Sáinz). Scientific names were checked with a taxonomic thesaurus incorporated in the database software (according to current trends in harvestmen nomenclature: Prieto 2003, 2008). Possible mistakes in geographic coordinates (format, localities within country/provincial boundaries), in the format or coherence of dates, or in ASCII anomalous characters were checked using automated routines with DARWIN_TEST (v3.2) software.

## Datasets

### Dataset description

**Object name:** Darwin Core Archive BOS Arthropod Collection of University of Oviedo (Spain): Opiliones

**Character encoding:** UTF-8

**Format name:** Darwin Core Archive format

**Format version:** 1.0

**Distribution:** http://www.gbif.es:8080/ipt/archive.do?r=bos-opi

**Publication date of data:** 2013-07-04

**Update police**: Annually when necessary to transmit data of new specimens kept at BOS Collection.

**Language:** Spanish

**Licenses of use:** This dataset [BOS Arthropod Collection of University of Oviedo (Spain): Opiliones (BOS-Opi)] is made available under the Open Data Commons Attribution License: http://www.opendatacommons.org/licenses/by/1.0/.

**DarwinCore elements**: The DarwinCore elements (http://purl.org/dc/terms/) included in the dataset published through the GBIF network describe the specimens’ data to several levels. These elements are: Record data: type (basisofrecord), DateLastModified, InstitutionCode, CollectionCode, CatalogNumber, Collector, IndividualCount, Sex, YearCollected, MonthCollected, DayCollected, Notes (with info about habitat in most of cases); Geographic data: Country, StateProvince, Locality (including municipality when available), MinimumElevation (meters), MaximunElevatium (meters), Latitude (decimalLatitude), Longitude (decimalLongitude), CoordinatePrecision (meters); Taxonomic data: Kingdom (Animalia all records), Phylum (Arthropoda all records), Class (Arachnida all records), Order (Opiliones all records), Family, Genus, Species (specificEpithet), ScientificNameAuthor (authorship of taxa name), ScientificName, Identified by, Yearidentified, Type status. Moreover, some DarwinCore elements were mapped to fixed values in the IPT as described in this data-paper: language, rights, rightsHolder, bibliographicCitation, references, datasetID, datasetName, ownerInstitutionCode.

## External datasets

### Dataset description

**Object name:** BOS Arthropod Collection of University of Oviedo (Spain): Opiliones

**Character encoding:** iso-8859-1

**Format name:** Darwin Core Archive

**Format version:** 1.0

**Distribution:** http://data.gbif.org/datasets/resource/15038

**Metadata language:** English

**Date of metadata creation:** 2013-06-12

**Hierarchy level:** Dataset
